# Updating Framingham CVD risk score using waist circumference and estimated cardiopulmonary function: a cohort study based on a southern Xinjiang population

**DOI:** 10.1186/s12889-022-14110-y

**Published:** 2022-09-09

**Authors:** Xue-Ying Sun, Ru-Lin Ma, Jia He, Yu-Song Ding, Dong-Sheng Rui, Yu Li, Yi-Zhong Yan, Yi-Dan Mao, Sheng-Yu Liao, Xin He, Shu-Xia Guo, Heng Guo

**Affiliations:** 1 Department of Public Health, Shihezi University School of Medicine, North 2th Road, Shihezi, Xinjiang 832000 China; 2 NHC Key Laboratory of Prevention and Treatment of Central Asia High Incidence Diseases, First Affiliated Hospital, School of Medicine, Shihezi University, ShiHezi, XinJiang, 832000, China

**Keywords:** Framingham risk score, Waist circumference, Estimated cardiorespiratory function, Model updating, Online risk calculator

## Abstract

**Purpose:**

To explore the association between waist circumference (WC), estimated cardiopulmonary function (eCRF), and cardiovascular disease (CVD) risk in southern Xinjiang. Update the Framingham model to make it more suitable for the southern Xinjiang population.

**Methods:**

Data were collected from 7705 subjects aged 30–74 years old in Tumushuke City, the 51st Regiment of Xinjiang Production and Construction Corps. CVD was defined as an individual's first diagnosis of non-fatal acute myocardial infarction, death from coronary heart disease, and fatal or non-fatal stroke. The Cox proportional hazards regression analysis was used to analyze the association between WC, eCRF and CVD risk. Restricted cubic spline plots were drawn to describe the association of the two indicators with CVD risk. We update the model by incorporating the new variables into the Framingham model and re-estimating the coefficients. The discrimination of the model is evaluated using AUC, NRI, and IDI metrics. Model calibration is evaluated using pseudo R^2^ values.

**Results:**

WC was an independent risk factor for CVD (multivariate HR: 1.603 (1.323, 1.942)), eCRF was an independent protective factor for CVD (multivariate HR: 0.499 (0.369, 0.674)). There was a nonlinear relationship between WC and CVD risk (nonlinear χ2 = 12.43, *P* = 0.002). There was a linear association between eCRF and CVD risk (non-linear χ2 = 0.27, *P* = 0.6027). In the male, the best risk prediction effect was obtained when WC and eCRF were added to the model (AUC = 0.763((0.734,0.792)); pseudo *R*^2^ = 0.069). In the female, the best risk prediction effect was obtained by adding eCRF to the model (AUC = 0.757 (0.734,0.779); pseudo *R*^2^ = 0.107).

**Conclusion:**

In southern Xinjiang, WC is an independent risk factor for CVD. eCRF is an independent protective factor for CVD. We recommended adding WC and eCRF in the male model and only eCRF in the female model for better risk prediction.

**Supplementary Information:**

The online version contains supplementary material available at 10.1186/s12889-022-14110-y.

## Introduction

Cardiovascular disease (CVD) is the leading cause of death and disease burden worldwide. It is one of the important public health problems to be solved urgently [1]. There are many traditional CVD risk factors, such as dyslipidemia, abnormal blood pressure, and obesity [2]. With the continuous exploration of non-traditional CVD risk factors, researchers discover more emerging CVD risk factors, such as Estimated Cardiorespiratory Fitness (CRF) [3], sleeping mode [4]. Researchers use CVD risk factors to establish a risk prediction model to assess individual CVD risk, which is an important measure for the primary prevention of CVD.

Framingham risk score is the most classic CVD risk prediction model and is widely used worldwide, which is based on the Framingham cohort [5]. Predictors of the Framingham model included age, systolic blood pressure (SBP), high-density lipoprotein cholesterol (HDL-C), total cholesterol (TC), smoking status, and history of diabetes. Framingham risk score does not incorporate some emerging, easily measurable indicators.

Xinjiang is located in the northwest of China and is a typical multi-ethnic inhabited area. Uyghurs account for 45.85% of the total population of Xinjiang. They are mainly distributed in southern Xinjiang and are the main resident population in rural areas of southern Xinjiang. Compared with the Han nationality, the Uyghur nationality has a unique lifestyle, dietary habits, and genetic characteristics. This population has a higher risk of CVD [6] and requires effective primary CVD prevention measures.

During the previous investigation, we found that obesity and abdominal obesity were risk factors for elevated blood pressure in remote rural areas of Xinjiang [7]. Waist circumference (WC) is a commonly used indicator that can better reflect the degree of obesity and abdominal obesity [8]. Similarly, in the previous survey, we found that fewer people in southern Xinjiang maintain the habit of exercising. In 2016, the American Heart Association proposed to pay attention to the importance of cardiorespiratory fitness in clinical practice, at least using non-exercise prediction equations for routine clinical assessment of cardiorespiratory fitness, A common indicator is Estimated Cardiorespiratory Fitness (eCRF) [9]. eCRF uses readily available clinical information to estimate the subject's cardiopulmonary exercise, such as age, gender, resting heart rate, and physical activity. Compared with CRF obtained through cardiopulmonary exercise testing, eCRF is less expensive and easier to obtain. Therefore, we hope to explore the association between WC, eCRF at baseline and CVD risk in the southern Xinjiang population. Then, we add these two risk factors to the Framingham model to obtain a more suitable model for the southern Xinjiang population. To facilitate the promotion and use of predictive models, we build an online CVD risk calculator based on the coefficients of the best model.

## Material and methods

### Study population

The subjects were adults aged ≥ 18 years who lived in Tumushuke City, 51st Regiment, Xinjiang Production and Construction Corps above 6 months from September 2016 to August 2021, with a median follow-up time of 4.97 years. We started this study in September 2016. This study adopts the stratified random cluster sampling method. In the early stage, the Xinjiang Uygur Autonomous Region was stratified according to the southern Xinjiang/northern Xinjiang, the corps area/the non-corps area. Finally, the southern Xinjiang and corps areas were selected. We selected the third division after the first cluster sampling. After the second cluster sampling, we selected the 51st regiment as our research site. We conducted a census of permanent residents ≥ 18 in the 51st regiment and took hospitals and communities as our study sites for questionnaires, anthropometric measurements, and blood sample collection. The Uyghurs are the main permanent residents in the southern Xinjiang region, the area where this study is carried out is the Uyghur inhabited area. Considering that the living environment of the southern Xinjiang region is similar, the Uyghurs have the same dietary habits, genetic backgrounds, and living habits, and the random sampling method was strictly followed in the field, so it can be regarded as representative of the Uyghur population in southern Xinjiang.

**Fig. 1:**
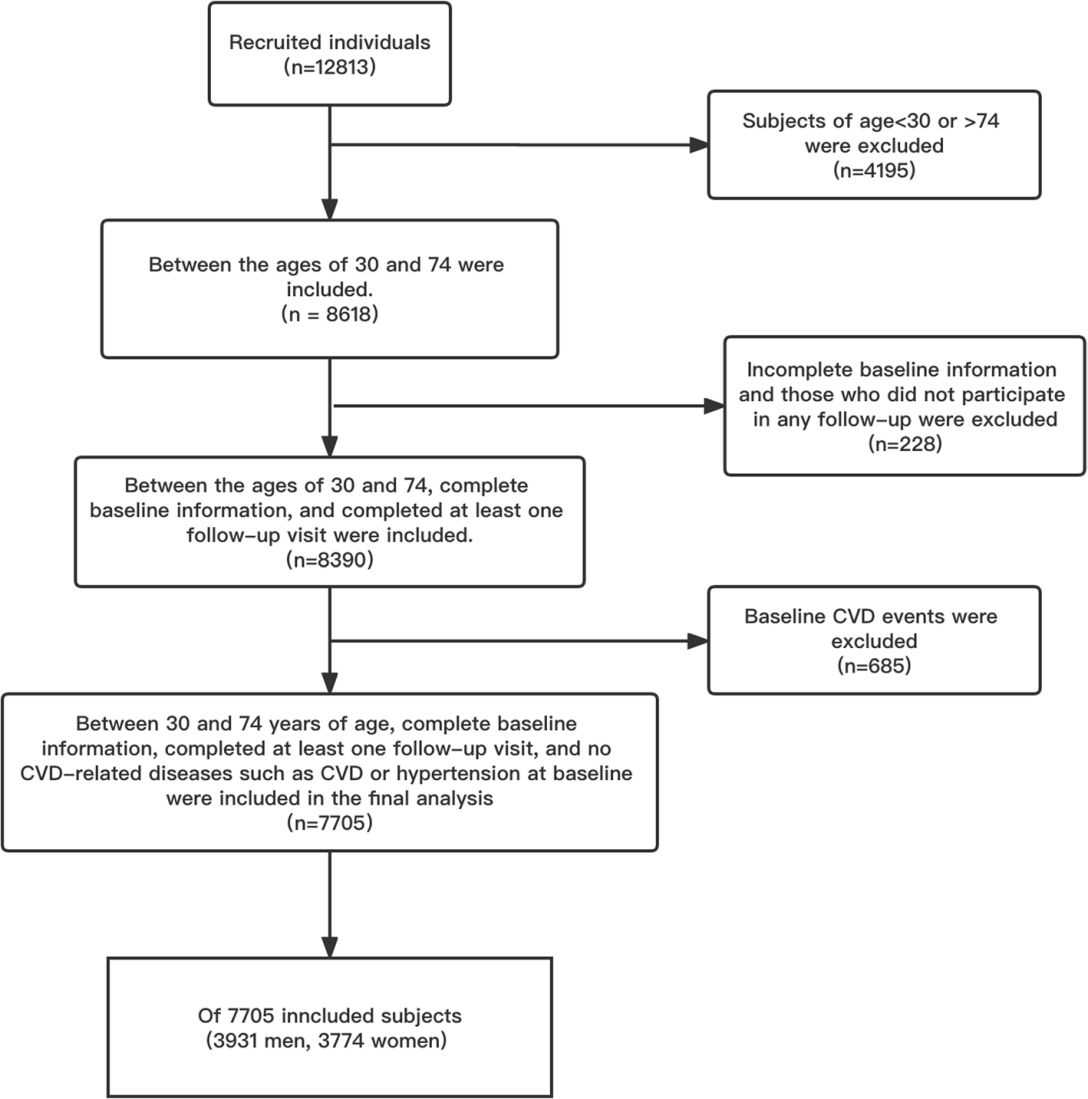
Flow chart of inclusion and exclusion of study population. Abbreviations: Adjusted for gender, age, educational status, career, marital status, exercise status, smoking, drinking, TC, and HDL. global χ2 = 626.68, *P* < 0.001; nonlinear χ2 = 12.43, *P* = 0.002. Cut-Point: WC = 82.42 cm

The participants aged 30–74 years were selected. They had no history of cardiovascular disease (CVD) at baseline. They had complete baseline information and participated in at least one follow-up visit throughout the follow-up period. Floating population, population with mental illness or intellectual disability, pregnant women and people with chronic kidney disease were excluded from this study. According to the inclusion and exclusion criteria of this study (Fig. 1), 7705 subjects aged 30–74 years were included in the final analysis.

### Questionnaire and follow-up

The epidemiological survey was carried out in the 51st Regiment of the Third Division of the Xinjiang Production and Construction Corps in 2016. The survey included a questionnaire, a collection of blood biochemical indicators, and a collection of physical indicators. And three follow-up surveys were conducted in 2019, 2020, and 2021 respectively. The follow-up survey content was consistent with the baseline survey content. The social security information, hospitalization information, and chronic disease information during the follow-up period were also collected.

Participants were interviewed face-to-face by standard questionnaires, which includes information on sociodemographic characteristics, medical history and lifestyle habits. All participants have lived in the rural areas of Southern Xinjiang for more than 6 months. Current smoking status was self-reported by the participants. Family history of CVD was defined as a parent or sibling with a history of coronary heart disease, myocardial infarction, or stroke.

The physical examination was conducted by professionally trained investigators. Waist circumference, which was measured with an inelastic tape measure, was defined as the midpoint between the lower rib and the superior border of the iliac crest at minimum breathing. The blood pressure of the participants was measured with electronic sphygmomanometers (OMRON HEM-7051, Omron (Dalian Co., Ltd.)). Each individual was measured twice with an interval of 30 s. The average was taken as the final blood pressure result.

A 5 ml fasting blood sample was collected from each subject. Blood glucose, high-density lipoprotein cholesterol, and total lipoprotein cholesterol were determined by the modified hexokinase enzymatic method using the Japanese Olympus AV2700 biochemical automatic analyzer in the Biochemical Laboratory of the First Affiliated Hospital of Shihezi University School of Medicine.

CVD events in the study cohort were determined from patients' hospital medical records, questionnaires, and social security records. Questionnaires were used to follow up with the subjects, and the disease information of the subjects was collected and checked with the hospital social security data and medical record information. If the subjects died during the follow-up period, the family members will be asked about the time of death, the place of death, and the cause of death, and then this information will be checked against the information records provided by the hospital.

All participants signed informed consent. This study was approved by the Ethics Committee of the First Affiliated Hospital of Shihezi University School of Medicine (No. SHZ2010LL01).

### CVD outcome definitions

In this study, CVD was defined as an individual's first diagnosis of non-fatal acute myocardial infarction, death from coronary heart disease, and fatal or non-fatal stroke.

Acute myocardial infarction was defined as an increase in biochemical markers of myocardial necrosis with ischemic symptoms, pathological Q waves, ST-segment elevation or depression, or coronary intervention. Coronary heart disease deaths include all fatal events due to myocardial infarction or other coronary death. Stroke was defined as an ischemic or hemorrhagic attack. If more than one CVD event occurred during follow-up, only the first CVD event was included as an outcome event.

### Framingham risk score equations

We use the Framingham CVD prediction model developed in 2008 for people aged 30–74 [5]. The model equations are as follows: Male = 1-(0.9431^exp (age *3.06117 + TC *1.12370–0.93263*HDL-C + 1.93303*SBP + 0.65451*smoking status + 0.57367*Diabetes-23.9802); Female = 1-(0.9747^exp (age *2.32888 + 1.20904*TC -0.70833*HDL-C + 2.76157*SBP + 0.52873*smoking status + 0.69154*Diabetes-26.1931). We performed a simple calibration of the Framingham model using means of risk factors in this population and risk of morbidity [10].

### Statistical analysis

Continuous variables that satisfy the normal distribution are described by the mean ± standard deviation. Continuous variables that do not satisfy the normal distribution are described by the median and interquartile range. Categorical variables are described by the sample size and percentage. Since the follow-up period of this study was five years, only the five-year CVD actual risk and the five-year predicted risk were calculated in this study.

Calculate eCRF using gender-specific equations. Female(METs) = 14.7873 + (age × 0.1159) – (age^2^ × 0.0017) – (BMI × 0.1534) – (waist circumference × 0.0088) – (resting heart rate × 0.364) + (physical activity [active vs inactive] × 0.5987) – (smoking [yes vs no] × 0.2994); eCRF in male(METs) = 21.2870 + (age × 0.1654) – (age^2^ × 0.0023) – (BMI × 0.2318) – (waist circumference × 0.0337) – (resting heart rate × 0.0390) + (physical activity [active vs inactive] × 0.6351) – (smoking [yes vs no] × 0.4263) [11]. BMI indicates body mass index (calculated as weight in kilograms divided by height in meters squared).

WC and eCRF were grouped by tertiles, with the lowest group serving as the reference group. The log-rank test was used to compare the risk of CVD morbidity among eCRF groups and WC groups. We performed pairwise comparisons among the three groups and used a Bonferroni-corrected P-value (*P* = 0.017) to ensure the accuracy of the log-rank between-group test results (https://www.graphpad.com/support/faq/after-doing-logrank-analysis-on-three-or-more-survival-curves-can-i-perform-multiple-tests-for-differences-between-pairs-of-curves/).

A univariate COX proportional hazards regression was used to analyze the association between WC, eCRF, and CVD risk. Age, educational status, career, marital status, exercise status, smoking, alcohol consumption, TC, and HDL-C were adjusted as confounders during multivariate COX proportional hazards regression analysis. Exploring the association between eCRF, WC, and CVD risk in this population using a restricted cubic spline with 4 knots, with knots equally distributed. We take the point in the restrictive cubic spline where the direction of HR change changes as a rough value for the change in the variable.

This study calibrated the Framingham original model by mean levels of risk factors and the five-year risk of CVD in this population. The WC and eCRF were added to the Framingham model for model adjustment. After introducing new risk factors, use bootstrap 1000 times to internally validate the model. The discrimination of the model is evaluated using AUC(Area Under Curve), NRI(Net Reclassification Index), and IDI(Integrated Discrimination Improvement) metrics. We calculated categorical NRI and continuous NRI separately, using 10% and 20% as risk cut-off points for categorical NRI. We use the Delong test to analyze whether there is a difference in AUC between the model after adding the new variable and the original model. Model calibration is evaluated using pseudo R^2^ values. High pseudo R^2^ values indicate better discrimination. We choose the model with the best predictive performance and build an online risk calculator based on the coefficients for each risk factor.

SPSS and R software were used for data analysis. JAVA Script was used to build an online risk calculator.

## Results

### Baseline characteristics

A total of 7705 participants were finally included in this study. The male participants accounted for 51.02% of the total participants (Fig. 1). According to Table 1, the average age of males and females was 44.0 ± 10.8 years and 43.2 ± 10.3 years, respectively. The WC and eCRF of the male were 95.5 ± 13.3 cm and 11.3 ± 1.52Mets, which were significantly higher than those of women (*P* < 0.001). In addition, among the male population, the number of smokers was 1141(29.03%) and the number of diabetic patients was 607(15.04%), which was significantly higher than those of females (*P* < 0.001). However, during the entire follow-up period, a total of 293 CVD events occurred in men, with a five-year CVD incidence rate of 7.5%. A total of 500 CVD events occurred in women, with a five-year CVD incidence rate of 13.2%. Females had a significantly higher risk of CVD than males (*P* < 0.001).

**Table 1:** Descriptive table of baseline characteristics of different genders in the study population

Characteristics	Male (*n* = 3931)	Female (*n* = 3774)
**Age, Mean (SD), y**	44 (10.8)	43.2 (10.3)
**SBP, Mean (SD), mmHg**	132.1 (19.7)	131.2 (21.3)
**Smoking, No. (%)**	1141 (29.03)	24 (0.64)
**Diabetes, No. (%)**	607 (15.4)	473 (12.5)
**HDL, Mean (SD), mg/dl**	26.1 (9.53)	27.6 (9.30)
**TC, Mean (SD), mg/dl**	87.2 (20.7)	85.8 (19.9)
**WC, Mean (SD), cm**	95.5 (13.3)	93.3 (13.9)
**eCRF, Mean (SD), Mets**	11.3 (1.52)	8.57 (1.10)
**Total person years**	18,543	17,239
**Incidence of CVD events within 5 years**	293	500
**5-year Kaplan–Meier CVD rate (%)**	7.5	13.2

**Abbreviations:** *SD* Standard Deviation, *SBP* Systolic blood pressure, *DBP* Diastolic blood pressure, *TC* Total cholesterol, *HDL-C* High density lipoprotein cholesterol, *Diabetes* Diabetes mellitus, *eCRF* Estimated cardiopulmonary function, *WC* Waist circumference, *CVD* Cardio vascular disease, *K-M* Kaplan- Meier analyze

### Log-rank test between different eCRF and waist circumference groups

Taking any group as the reference, the *P*-values were all less than 0.001 when comparing the two groups. Therefore, there is a significant difference between the groups for WC and eCRF (Table 2, Supplement 2).

**Table 2:** Log-rank test between waist circumference and eCRF group

		**Low**		**Medium**		**High**	
		**χ**^**2**^	***P-value***	**χ**^**2**^	***P-value***	**χ**^**2**^	***P-value***
**WC**	**Low, < 89 cm**	-	-	17.22	< 0.001	112.16	< 0.001
	**Medium, 89 cm ≤ x < 100 cm**	17.22	< 0.001	-	-	46.38	< 0.001
	**High, ≥ 100 cm**	112.16	< 0.001	46.38	< 0.001	-	-
**eCRF**	**Low, < 8.93**	-	-	101.25	< 0.001	256.70	< 0.001
	**Medium, 8.93 ≤ x < 10.87**	101.25	< 0.001	-	-	43.85	< 0.001
	**high, ≥ 10.87**	256.70	< 0.001	43.85	< 0.001	-	-

### Univariate COX proportional hazards regression analysis

Taking the lowest group as the reference group, the HR value of the middle WC group was 1.496 (95%CI: 1.233, 1.814), and the HR value of the high waist circumference group was 2.567 (95%CI: 2.140, 3.080). When WC increased, the risk of CVD increased. Similarly, the HR value of the moderate eCRF group was 0.440 (95%CI: 0.375, 0.517), and the HR value of the high eCRF group was 0.209 (95%CI: 0.170, 0.259). When eCRF increased, the risk of cardiovascular disease decreased (Table 3, Supplement 2).

**Table 3:** Univariate COX proportional hazards regression analysis results table

			**SE**	**OR**	**95%CI**	***P-value***
**WC**	**Low, < 89 cm**	1	1	1	-	-
	**Medium, 89 cm ≤ x < 100 cm**	0.413	0.099	1.511	(1.245,1.835)	< 0.001
	**High, ≥ 100 cm**	0.942	0.094	2.566	(2.136,3.082)	< 0.001
**eCRF**	**Low, < 8.93**	1	1	1	-	-
	**Medium, 8.93 ≤ x < 10.87**	-0.821	0.082	0.440	(0.375,0.517)	< 0.001
	**high, ≥ 10.87**	-1.563	0.108	0.209	(0.170,0.259)	< 0.001

**Abbreviations:** *SE* Standard error, *CI* Confidence interval

### Multivariate COX proportional hazards regression analysis

After adjusting for confounding factors, the OR values of the middle WC group and the high WC group were 1.315 (95%CI:1.082, 1.598) and 1.890 (95%CI: 1.566, 2.281). WC is an independent risk factor for CVD. After adjusting for confounding factors, the OR values of the eCRF medium group and eCRF high group were 0.704 (95%CI: 0.580, 0.855) and 0.499 (0.369, 0.674). eCRF is an independent CVD protective factor (Table 4).

**Table 4:** Multivariate COX proportional hazards regression analysis results

			**SE**	**OR**	**95%CI**	***P-value***
**WC**	**Low, < 89 cm**	1	1	1	-	-
	**Medium, 89 cm ≤ x < 100 cm**	0.183	0.100	1.201	(0.986,1.461)	0.068
	**High, ≥ 100 cm**	0.472	0.098	1.603	(1.323,1.942)	< 0.001
**eCRF**	**Low, < 8.93**	1	1	1	-	-
	**Medium, 8.93 ≤ x < 10.87**	-0.351	0.099	0.704	(0.580,0.855)	0.001
	**high, ≥ 10.87**	-0.696	0.154	0.499	(0.369,0.674)	< 0.001

**Abbreviations:** Every analysis adjusted for gender, age, educational status, career, marital status, exercise status, smoking, drinking, TC, HDL

*SE* Standard error, *CI* Confidence interval

### Associations between eCRF, waist circumference, and CVD risk

**Fig. 2:**
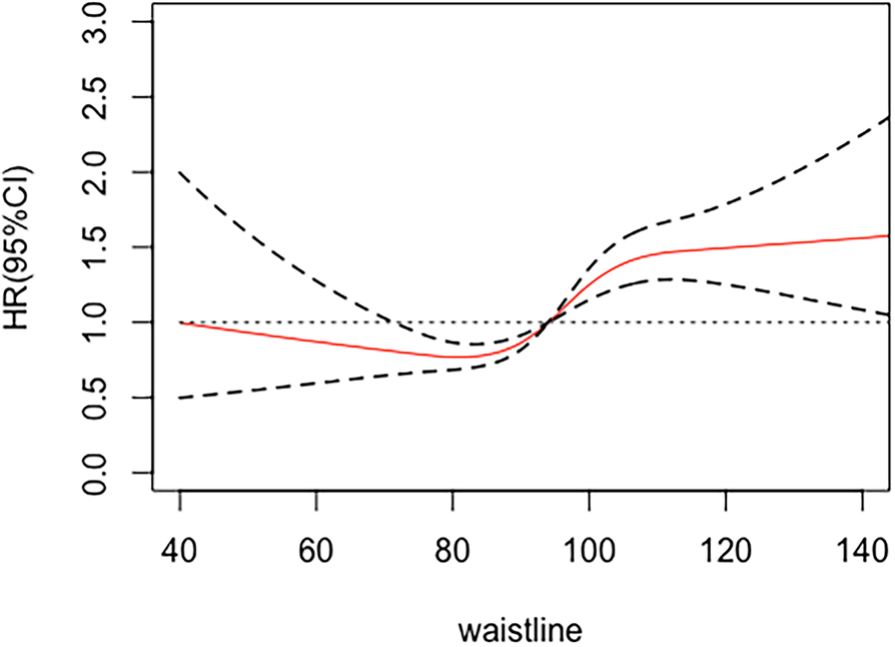
Restricted cubic spline plot of waist circumference and CVD risk. Abbreviations: Adjusted for gender, age, educational status, career, marital status, exercise status, smoking, drinking, TC, and HDL. global χ2 = 634.78, *P* < 0.001; nonlinear χ2 = 0.27, *P* = 0.6027

The association between WC and CVD risk was S-type (global χ2 = 626.68, *P* < 0.001; nonlinear χ2 = 12.43, *P* = 0.002), when the WC was less than 82.42 cm, the risk of CVD decreased with the increase of WC. When the WC was higher than 82.42 cm, the risk of CVD increased with the increase of WC (Fig. 2).

**Fig. 3:**
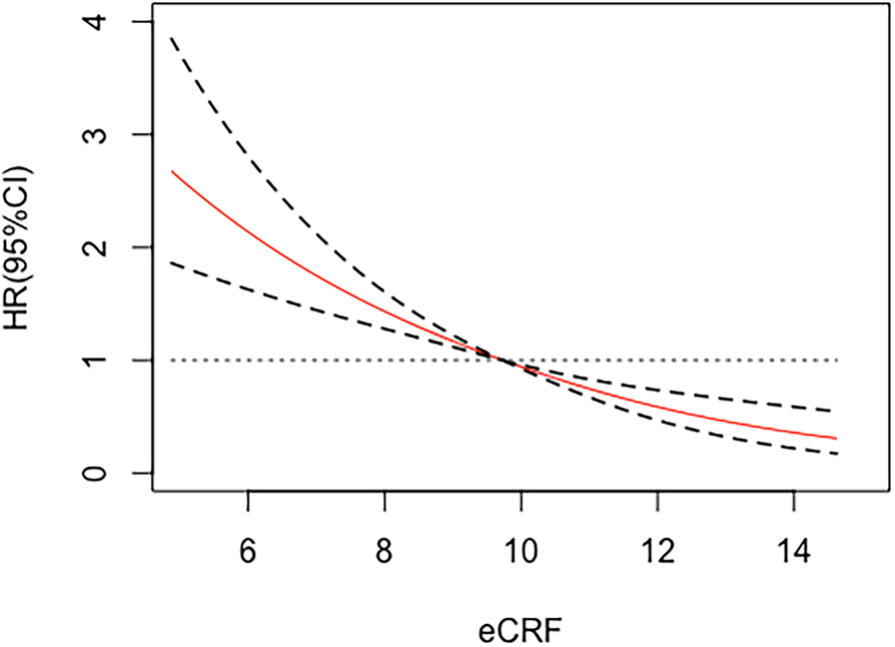
Restricted cubic spline plot of eCRF and CVD risk

There was a linear association between eCRF and CVD risk (global χ2 = 634.78, *P* < 0.001; nonlinear χ2 = 0.27, *P* = 0.603). Figure 3 shows the association between eCRF and the risk of CVD. It can be seen that with the increase of eCRF, the risk of CVD decreases (Fig. 3).

### Comparison of models after adding new variables

In the male population, after including WC, the model discrimination increased significantly by 7.9% (NRI = 0.079, *P* = 0.005). After including eCRF, the model discrimination increased significantly by 9.4% (NRI = 0.095, *P* = 0.002). After adding both WC and eCRF variables, model discrimination was significantly improved by 10% (NRI = 0.070, *P* = 0.015). When adding WC and eCRF at the same time, the pseudo R^2^ of the model is 0.069, a relatively high calibration level is obtained (Table 5).

**Table 5:** Comparison of discrimination and calibration among male prediction model

	**AUC**	**95%CI**	***P-value***	**NRI**	***P-value***	**cNRI**	***P-value***	**IDI**	***P-value***	**pseudo R**^**2**^
**Model1**	0.725	(0.693, 0.755)	-	1	-	-	-	1	-	NE
**Model2**	0.760	(0.730,0.789)	< 0.001	0.079	0.005	0.128	0.033	0.008	0.197	0.068
**Model3**	0.763	(0.734,0.792)	< 0.001	0.094	0.002	0.126	0.036	0.011	0.096	0.069
**Model4**	0.763	(0.734,0.792)	< 0.001	0.100	0.001	0.165	0.006	0.012	0.081	0.069

**Abbreviations:** Model1 is the Framingham model corrected with the data of this population. Model2 is the model with only waist circumference variables added. Model3 is the model with only eCRF added. Model4 is the model with both eCRF and waist circumference variables added

*NE* Not estimated

In the female population, the addition of eCRF increased the AUC value by 0.021, which was the same as the change in AUC values obtained by adding both WC and eCRF. In terms of calibration degree, the calibration level was relatively high when only eCRF was added, and the pseudo *R*^2^ was 0.107, which was the same as that after adding waist circumference and eCRF at the same time (Table 6).

**Table 6:** Comparison of discrimination and calibration among female prediction model

	**AUC**	**95%CI**	***P-value***	**NRI**	***P-value***	**cNRI**	***P-value***	**IDI**	***P-value***	**pseudo R**^**2**^
**Model1**	0.736	(0.710, 0.756)	-	1	-	-	-	1	-	NE
**Model2**	0.754	(0.728,0.773)	0.001	0.008	0.704	0.114	0.016	0.006	0.276	0.106
**Model3**	0.757	(0.734,0.779)	< 0.001	0.022	0.283	0.073	0.127	0.010	0.063	0.107
**Model4**	0.757	(0.734,0.779)	< 0.001	0.021	0.322	0.077	0.105	0.010	0.061	0.107

**Abbreviations:** Model1 is the Framingham model corrected with the data of this population, Model2 is the model with only waist circumference variables added, Model3 is the model with only eCRF added, and Model4 is the model with both eCRF and waist circumference variables added

*NE* Not estimated

The male population obtained the best prediction effect by including both eCRF and waist circumference variables. The female population could obtain the best prediction effect by including only the eCRF.

### Online risk calculator

To facilitate the application of CVD risk assessment, an online risk calculator was built according to each model coefficient. Users can enter personal information online to obtain the individual's risk of CVD in the next five years (Supplement 1).

## Discussion

This study analyzed the association between eCRF, WC, and the risk of CVD in the southern Xinjiang population. Then, we put WC and eCRF into the Framingham model for model adjustment. The results of the study showed that eCRF was negatively correlated with the risk of CVD in the southern Xinjiang population, and WC was positively correlated with the risk of CVD in the southern Xinjiang population. This association remained significant after adjustment for confounders. Further analysis found that with the increase of eCRF, the risk of CVD in this population decreased. However, when the WC was lower than 82.42 cm, the risk of CVD incidence decreased with the increase in waist circumference. When the WC was higher than 82.42 cm, the risk of CVD incidence showed an upward trend. After incorporating eCRF and WC into the Framingham model for coefficient adjustment, the discrimination and calibration of the new model were improved. Therefore, to obtain a better prediction effect, we suggested that both eCRF and WC variables should be included in the male model. To keep the model lean while being efficient, we recommended including only the eCRF variable in the model for the female population.

At present, there is a large amount of epidemiological evidence showing that obesity is associated with CVD, obesity can increase the risk and mortality of CVD [12]. Abdominal obesity, as a special form of obesity, is also closely related to the morbidity and mortality risk of CVD [13]. Some researchers have pointed out that patients should be educated that monitoring WC can be effective in preventing CVD [14]. In 2019, a study from Europe noted that changes in WC were associated with a higher risk of death from CVD in men [15]. In 2021, a study from China proposed that for the elderly population, increased WC may increase the risk of CVD mortality, and the dose–response relationship between baseline WC and CVD mortality is U-shaped/J-shaped [16]. This is consistent with our findings. A previous study on ethnic minority populations in rural areas of southern Xinjiang showed that maintaining a normal WC can effectively prevent the occurrence of coronary heart disease [17]. In 2022, some researchers suggest that WC should be considered in clinical practice as a simple marker of abdominal obesity, in combination with cardiorespiratory fitness, overall diet quality, and reported physical activity levels, to improve the ability to differentiate health risks in overweight/obese individuals [18]. While these are consistent with our findings, there are different voices. In 2021, some researchers proposed that although the risk of visceral obesity is always associated with adult CVD, this association remains controversial in the elderly population [19].

Cardiorespiratory fitness (CRF) refers to the ability of the cardiorespiratory system to supply oxygen to skeletal muscles during exercise, and regular physical activity improves the health of the cardiorespiratory system through physiological means [20]. CRF is an independent risk factor for CVD morbidity and mortality [21] eCRF is a method for estimating cardiorespiratory fitness through a non-exercise method. Studies have shown that eCRF has a good association with CRF measured by exercise testing [22]. Some researchers have proposed that the use of routinely collected information to obtain eCRF can provide a valid indication of health status [23]. A 2020 study from Taiwan suggests that routine assessment of eCRF in clinical practice may be useful for CVD prevention [24]. Results of a meta-analysis showed that eCRF is independently and inversely associated with the risk of cardiovascular mortality and all-cause mortality in the general population, eCRF may have potential as a valid and practical risk prediction tool in epidemiological or population-based studies [25]. In 2021, a study in Japan indicated that eCRF could be used to estimate cardiorespiratory function in a population without physical activity measurements, thereby further assessing CVD risk in this population [26]. In 2022, a study in China found that eCRF was inversely associated with CVD mortality risk [27]. The above results are consistent with our findings.

The correction and update of the model include five categories. In addition to the adjustment of the coefficients and intercepts of the model, it also includes re-estimating the coefficients using local population data or re-estimating the model coefficients after adding new variables [28]. In 2020, researchers recalibrated and re-estimated the Framingham and PCE equations using the Austrian Health Screening Program population information, the results showed that the re-estimation substantially improved the calibration of all Eqs[29] 27. In the same year, a result of adjusting and re-estimated the SCORE model in Eastern Europe showed that after re-estimated of the adjusted model with new predictors such as education, occupation, and stress, a better prediction effect was obtained [30]. In 2020, the results of a study on a Latino population showed that adding the CRF metric to the Framingham, SCORE, and PCE equations improved the predictive power of all three models [31]. In 2022, a Korean study added eCRF to the Framingham Risk Score and Mortality Score, the results showed that the discriminative ability of the model improved after adding eCRF [32]. The above results show that incorporating new risk factors and readjusting the model can lead to better predictions. These findings are consistent with our findings.

The advantages of this study are as follows. Firstly, the study population is representative. Then, the new variables included are easy-to-obtain variables, which can improve the prediction performance without increasing the detection burden. Finally, this study describes the association between eCRF and CVD risk in the southern Xinjiang Uyghur population.

This study also has some limitations. Firstly, the follow-up time is relatively short. Second, Uyghur-specific risk factors, such as sleep habits, dietary patterns, and history of parasitic diseases were not included. Similarly, more emerging CVD risk factors, especially female-specific CVD risk factors, such as gravidity, parity, and adverse pregnancy history were not included. Thus, further studies should include more extensive information on potential CVD influencing factors. Finally, there is no external validation of the model after adding new variables. These models should be externally validated in other populations in further studies. 

## Supplementary Information

**Additional file 1:** **Supplement 1.** **Supplement Table1.** Updated Framingham risk prediction model coefficients reestimated bygender.

**Additional file 2:** **Supplement 2.** **Figure S1.** Survival curve of cumulative incidence of CVDamong different WC groups. **Figure S2**. Survival curve of cumulative incidence of CVD among different eCRF groups.

## Data Availability

The datasets used during the current study are available from the corresponding author on reasonable request. The Chinese questionnaire copy may be requested from the authors.
