# Supplementary material for: Updating Framingham CVD risk score using waist circumference and estimated cardiopulmonary function: a cohort study based on a southern Xinjiang population
Source: BMC Public Health. 2022 Sep 9;22:1715. doi: 10.1186/s12889-022-14110-y (PMC9463829; doi:10.1186/s12889-022-14110-y)
Supplement: Supplementary file 1 — Additional file 1: Supplement 1. Supplement Table1. Updated Framingham risk prediction model coefficients reestimated bygender. [file 12889_2022_14110_MOESM1_ESM.pdf]

Supplement 1

We built an online risk calculator using the recalculated coefficients, which can be accessed at http://172.20.10.3:8080/. It has not yet been deployed on the server, and will be able to be accessed outside the local area network after completion. Here are the recalculated coefficients(Supplement Table 1):

Supplement Table 1. Updated Framingham risk prediction model coefficients re-estimated by gender

**coefficient**	**Male**	**Female**
Age_ln	2.3626	2.1287
TC_ln	-0.1754	-0.1846
HDL_ln	-0.7981	-0.8494
SBP_ln	2.0406	2.0069
Smoking status (0, no smoking)	-0.2142	-0.2475
DM	0.0663	0.1293
eCRF_ln	0.3474	-1.1457
WC_ln	-1.2921	NE
Five-year survival rate	0.925	0.868

Abbreviations: TC, Total cholesterol; HDL-C, High density lipoprotein cholesterol; SBP, Systolic blood pressure; DM, Diabetes mellitus; eCRF, Estimated cardiopulmonary function; WC, waist circumference; CVD, Cardio vascular disease
