# Supplementary material for: Updating Framingham CVD risk score using waist circumference and estimated cardiopulmonary function: a cohort study based on a southern Xinjiang population
Source: BMC Public Health. 2022 Sep 9;22:1715. doi: 10.1186/s12889-022-14110-y (PMC9463829; doi:10.1186/s12889-022-14110-y)
Supplement: Supplementary file 2 — Additional file 2: Supplement 2. Figure S1. Survival curve of cumulative incidence of CVDamong different WC groups. Figure S2. Survival curve of cumulative incidence of CVD among different eCRF groups. [file 12889_2022_14110_MOESM2_ESM.pdf]

**Supplement 2**

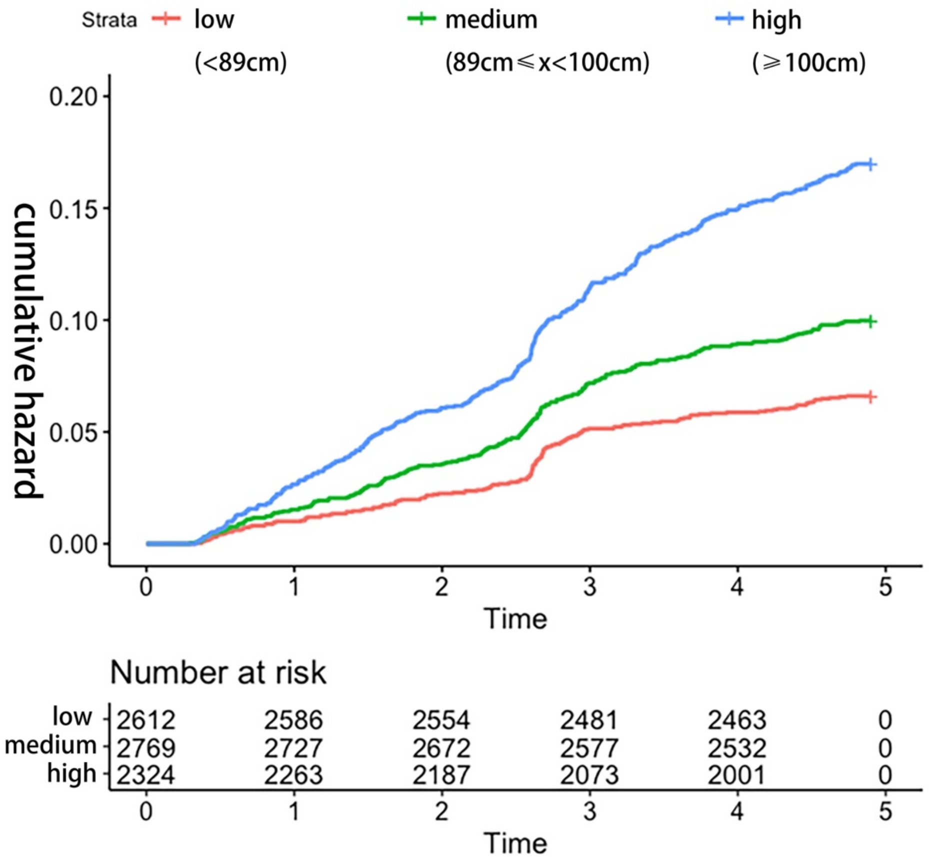


S1 Survival curve of cumulative incidence of CVD among different WC groups

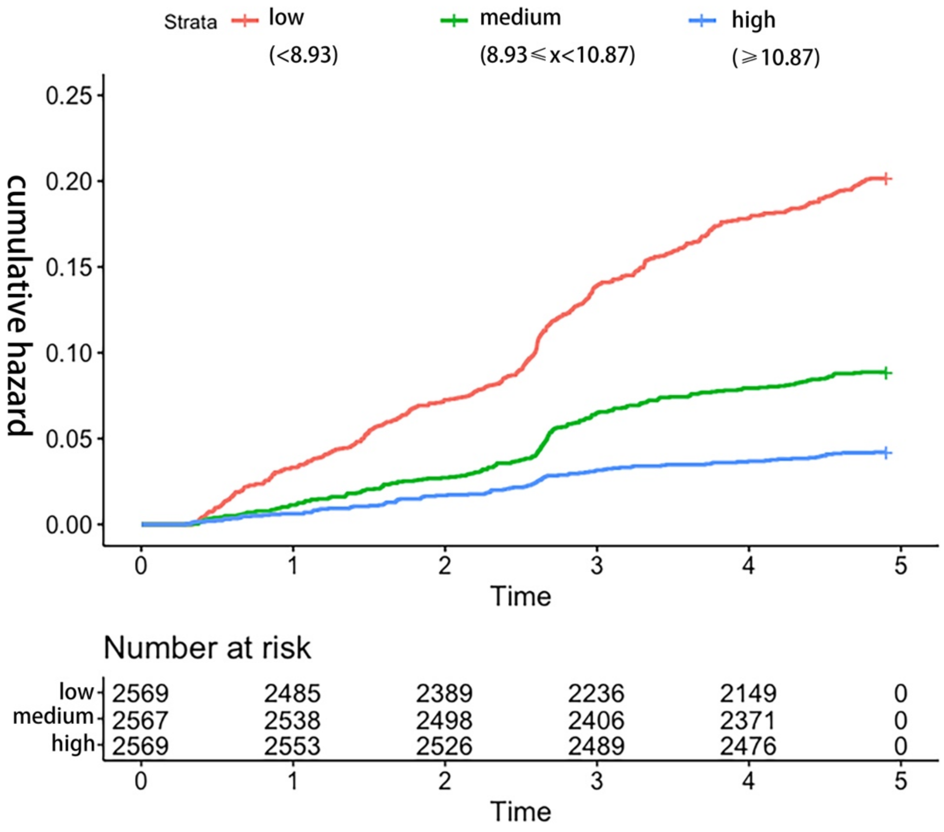


S2 Survival curve of cumulative incidence of CVD among different eCRF groups
